# The biological function and clinical significance of SF3B1 mutations in cancer

**DOI:** 10.1186/s40364-020-00220-5

**Published:** 2020-09-03

**Authors:** Zhixia Zhou, Qi Gong, Yin Wang, Mengkun Li, Lu Wang, Hongfei Ding, Peifeng Li

**Affiliations:** 1 Institute for Translational Medicine, The Affiliated Hospital of Qingdao University, College of Medicine, Qingdao University, Qingdao, 266012 People’s Republic of China; 2 The Second Clinical Medical College of Qingdao University, Qingdao, 266042 People’s Republic of China

**Keywords:** SF3B1, Mutation, Cancer, RNA splicing

## Abstract

Spliceosome mutations have become the most interesting mutations detected in human cancer in recent years. The spliceosome, a large, dynamic multimegadalton small nuclear ribonucleoprotein composed of small nuclear RNAs associated with proteins, is responsible for removing introns from precursor mRNA (premRNA) and generating mature, spliced mRNAs. SF3B1 is the largest subunit of the spliceosome factor 3b (SF3B) complex, which is a core component of spliceosomes. Recurrent somatic mutations in *SF3B1* have been detected in human cancers, including hematological malignancies and solid tumors, and indicated to be related to patient prognosis. This review summarizes the research progress of *SF3B1* mutations in cancer, including *SF3B1* mutations in the HEAT domain, the multiple roles and aberrant splicing events of *SF3B1* mutations in the pathogenesis of tumors, and changes in mutated cancer cells regarding sensitivity to SF3B small-molecule inhibitors. In addition, the potential of *SF3B1* or its mutations to serve as biomarkers or therapeutic targets in cancer is discussed. The accumulated knowledge about *SF3B1* mutations in cancer provides critical insight into the integral role the SF3B1 protein plays in mRNA splicing and suggests new targets for anticancer therapy.

## Background

Of the 3.3 billion base pairs of haploid DNA in the human genome, approximately 20,000 protein-coding genes have been identified by the Encyclopedia of DNA Elements (ENCODE) project [1]. However, the number of protein-coding genes is surprisingly low given the proteomic complexity, as the number of protein isoforms expressed from this gene set has been estimated to be at least 5–10-fold higher [2–4]. The generation of protein diversity is primarily due to the process of precursor mRNA (premRNA) splicing, which is controlled by a complex regulatory system that consists of an enormous number of sequence elements and trans-acting splicing factors; therefore, it is not surprising that the mRNA splicing machinery is susceptible to mutations and that these mutations are implicated in many human diseases, including cancer [5, 6]. Indeed, genome-wide studies have revealed more than 15,000 tumor-associated splice variants in a wide variety of cancers [7, 8].

Recently, the most interesting mutations detected in human cancer were found to target components of the spliceosome involved in the mRNA-splicing process, as indicated by genomic DNA analysis of a variety of human tumors studied through the Cancer Genome Project. One of the most exciting discoveries has been recurring somatic mutations in genes encoding 3 splice-site recognition protein components and serine/arginine-rich (SR) splicing factors, which were initially discovered in myelodysplastic syndrome (MDS) in 2011 and later reported in other hematological malignancies, including solid tumors [9–13]. Spliceosome mutations in cancers have highlighted the importance of the spliceosome pathway as a direct player in carcinogenesis and led to questions regarding the functional roles and molecular mechanisms of these mutations [14].

In this review, we describe spliceosome-associated transcript processing and its impact on disease. Moreover, we focus on one of the frequently mutated spliceosome proteins: cancer-related splicing factor 3b subunit 1 (SF3B1). We mainly summarize the distribution of mutations in *SF3B1*, mutant expression in tumors and its prognostic value. In particular, we discuss the functional consequences of *SF3B1* mutation in tumors, with multiple roles in tumor pathogenesis, aberrant splicing events, and changes in sensitivity to SF3B small-molecule inhibitors. The potential value of *SF3B1* or its mutation as a novel cancer therapeutic target and marker that is more sensitive to spliceosome inhibitors is also described. Finally, we explore the options available for future research on the biological function and clinical significance of *SF3B1* mutations in cancer.

## Precursor mRNA (premRNA) splicing

Precursor mRNA splicing is an essential step in the posttranscriptional regulation of gene expression and is a process that involves the removal of noncoding sequences (introns) from premRNA and the ligation of coding sequences (exons) to form mRNA. PremRNA splicing is catalyzed by the spliceosome, a complex consisting of 5 small nuclear RNAs (snRNAs) that associate with proteins to form particles termed small nuclear ribonucleoproteins (snRNPs) [15–17]. To date, two types of spliceosomes with unique compositions have been characterized: U2-dependent (major) spliceosomes and U12-dependent (minor) spliceosomes. The former spliceosome has been found in all eukaryotes and consists of the U1, U2, U5, and U4/U6 snRNPs and numerous proteins. Each U1, U2, and U5 snRNP has a single snRNA and several proteins; the U4 and U6 snRNPs have 2 snRNAs and several proteins. This spliceosome catalyzes the vast majority of transcript splicing events, removing the most commonly encountered class of introns (U2-type introns) (more than 99% in humans) [18, 19]. In contrast, the U12-dependent spliceosome is found only in a number of organisms, and acts on U12-type introns (less than 1% of introns in humans) [20, 21]. The difference between U2-type and U12-type introns is in the consensus splice-site sequence. The U12-dependent spliceosome also consists of 5 snRNPs, U11, U12, U5, and U4atac/U6atac snRNPs [22, 23].

**Fig. 1:**
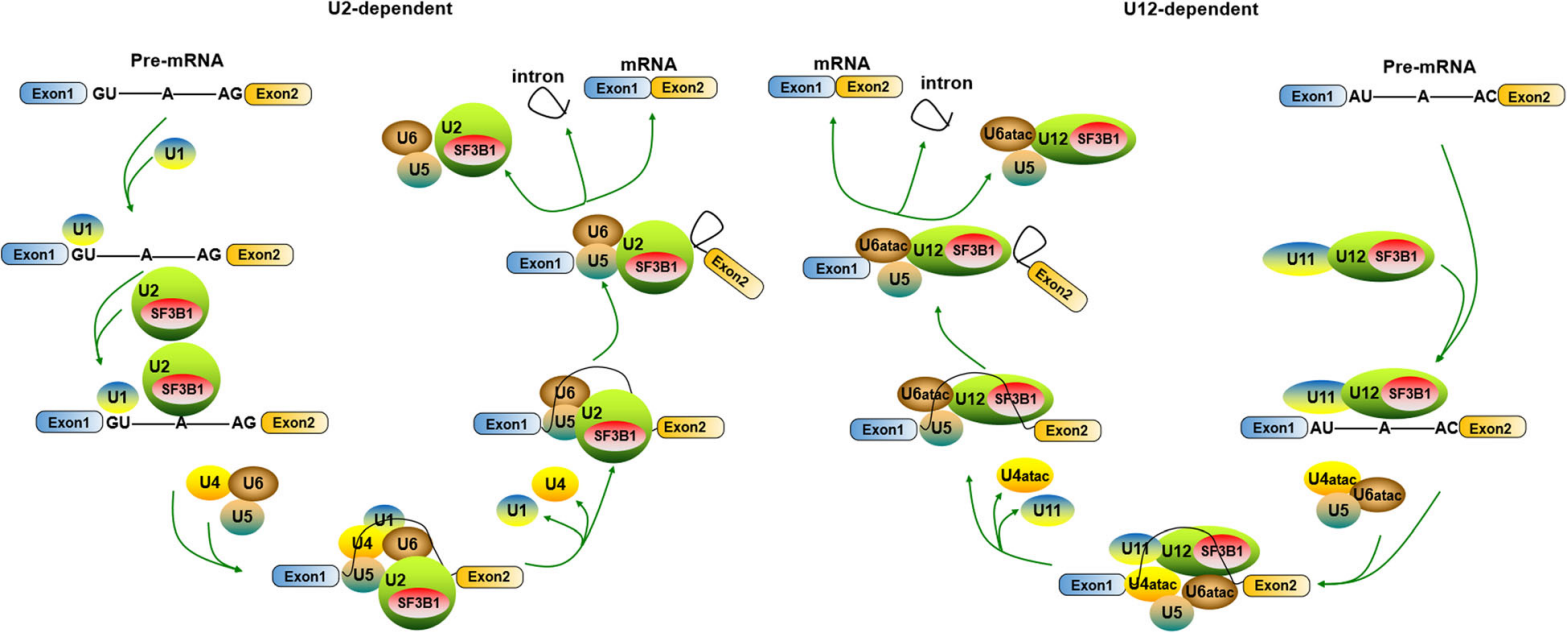
SF3B1 functions in the stepwise assembly of the U2- and U12-dependent spliceosomes. There are two types of spliceosomes: U2-dependent spliceosomes (left) and U12-dependent spliceosomes (right). Assembly of the U2-dependent spliceosome consists of 5 snRNPs, U1, U2, U5, and U4/U6 snRNPs; the U12-dependent spliceosome also consists of 5 snRNPs: U11, U12, U5, and U4atac/U6atac snRNPs. The difference between the two spliceosomes is in the consensus splice-site sequences, namely, U2-type or U12-type premRNA introns. SF3B1 is shared in the core components between the two spliceosomes and plays a key role in the recognition and selection of the branch site (BS) by interacting with premRNA in a sequence-independent manner, reinforcing stability during U2 (or U12) snRNA/BS interaction

The stepwise interactions between premRNA and both U2- and U12-dependent spliceosome snRNPs are highly ordered, as shown in Fig. 1. Briefly, assembly of the U2-dependent spliceosome is initiated by interaction of the U1 snRNP with the 5′ splice site, which includes a GU, leading to the formation of the E complex. Then, the U2 snRNP binds to the branch site (BS) to generate the A complex or the prespliceosome. When the U5 and U4/U6 snRNPs interact with the A complex, the B complex is generated. U4/U6 base-pairing interaction is disrupted, and U6 displaces U1 snRNA, which then binds to the 5′ splice site. After B complex rearrangement, a catalytically active B* complex is produced through U1 and U4 snRNP dissociation. The mRNA is released after completion of the first (the C complex is formed) and second catalytic steps of splicing, in which the intron is removed, and the spliceosome dissociates to be recycled for new premRNA splicing [24, 25]. Assembly of the U12-dependent spliceosome is similar to that of the U2-dependent spliceosome because U11, U12 and U4atac/U6atac snRNPs are functional analogs of the snRNPs U1, U2 and U4/U6. U12-dependent spliceosome assembly also involves sequential formation of the A, B, B*, and C complexes, but the earliest E complex formation step does not occur [21, 23]. The differences in the premRNA splicing mechanism mediated by the U2- and U12-dependent spliceosomes appear to involve only early intron recognition events and not the catalytic process.

A large, diverse and dynamic protein has been found that interacts with snRNAs to form snRNPs within the spliceosome. Although the U2- and U12-dependent spliceosomes differ in their snRNA composition, they share many proteins [26–29]. For example, all of the subunits of the protein complex SF3B, namely, SF3B155/SF3B1, SF3B145, SF3B130, SF3B49, SF3B14a/p14, SF3B14b and SF3B10, are the same [30]. SF3B contributes a molecular mass of ~ 450 kDa to each snRNP, and it has been demonstrated to play a key role in the recognition and selection of the branch site (BS) during splicing by interacting with the premRNA at or near the BS in a sequence-independent manner, reinforcing stability during the U2 snRNA/BS interaction [31]. In addition, numerous splicing factors, including an array of regulatory elements and proteins, participate in premRNA splicing events involving the two types of spliceosomes, such as exonic splicing enhancer (ESE), exonic splicing silencer (ESS), intronic splicing enhancer (ISE), intronic splicing silencer (ISS), SR proteins, heterogeneous nuclear ribonucleoproteins (hnRNPs), and others [5, 17, 32–34]. Thus, although premRNA splicing is traditionally considered to involve separate and sequential processes, it is difficult for spliceosomes and associated proteins to detect specific splice sites in a vast RNA pool. This complexity makes the premRNA splicing machinery susceptible to sequence polymorphisms and deleterious mutations, some of which eventually lead to diseases, the number of which is growing [5]. Therefore, some specific mutations or polymorphisms of premRNA splicing factors have become important diagnostic markers and therapeutic targets in human diseases.

## Alternative splicing

Two different modes of splicing have been defined: constitutive splicing and alternative splicing. Constitutive splicing is the process of removing introns from premRNA and joining the exons together to form a mature mRNA sequence. Alternative splicing is the process by which the exons are either retained or targeted for removal in different combinations to yield a diverse array of mRNAs from a single premRNA [35]. There are several distinct patterns of alternative splicing, including cassette exons (in which one or more exons are either skipped or included), alternative 5′ splice sites, intron retention, mutually exclusive exons, alternative 3′ splice sites, and complex splicing patterns [36]. More than 90% of human genes produce transcripts that are alternatively spliced, and 60% of the splice variants encode distinct protein isoforms with unique cellular functions or properties [37–39]. Thus, alternative splicing plays important biological roles in the proliferation, differentiation and/or development of cells. In humans, the regulation of alternative splicing is tightly controlled during normal biological events [40]. Misregulation of alternative splicing can lead to the production of aberrant protein isoforms, which may contribute to serious diseases, including cancers. Thus, an in-depth investigation of alternative splicing regulation has become the trend to understand the mechanisms of human diseases.

## Splicing mutations in human disease

Disease-related mutations can affect splicing by altering splice site sequences, splicing regulatory sequences, or genes of the splicing machinery itself (i.e., spliceosome mutations) [41, 42], and mutations of the splice-site sequences or of the splicing regulatory sequences have been documented in a variety of human diseases. For example, mutations in the splice-site sequences of the *HBB* (hemoglobin, beta) gene lead to abnormal splicing of *HBB* and defective synthesis of its protein β-globin in human β^+^-thalassemia [43–45]. In human multisystem proteinopathy and amyotrophic lateral sclerosis (ALS), mutations in the prion-like domains of hnRNPA2B1 and hnRNPA1 occur [46]. An array of mutations in splicing cis-acting sequences include those of *LKB1* (liver kinase B1), *KIT* (v-kit Hardy-Zuckerman 4 feline sarcoma viral oncogene homolog), *CDH17* (cadherin 17), *KLF6* (Kruppel-like factor 6) and *BRCA1* (breast cancer gene 1) in many types of cancers [47, 48].

In addition to mutations that alter precursor RNA sequence elements that regulate splicing, components of the spliceosome machinery have been shown to be dysregulated in human disease. An example discovered relatively early is that mutations in tri-snRNP (small ribonucleoprotein), a preassembled complex of U4 snRNA hybridized to U6 (U4/U6 or U4atac/U6atac) that also contains U5 (U4/U6 or U5) and associated proteins, can cause an autosomal dominant form of retinitis pigmentosa (adRP) [6, 34]. Since 2011, recurrent somatic mutations have been identified in a number of spliceosome components in human malignancies through the Cancer Genome Project, such as U2AF1 (U2AF35), SRSF2 (SC35), SF3B1 (SF3B155 or SAP155), and ZRSR2 (URP) [13]. These spliceosome component mutations indicates somatic mutations are an important molecular mechanism underlying splicing deregulation in diseases. In this review, we discuss recently discovered examples of disease-linked spliceosome mutations in *SF3B1* that are shared in the core comments between the two spliceosomes (Fig. 1).

## *SF3B1* mutations in cancer

### Distribution of mutations in the molecular architecture of SF3B1

**Fig. 2:**
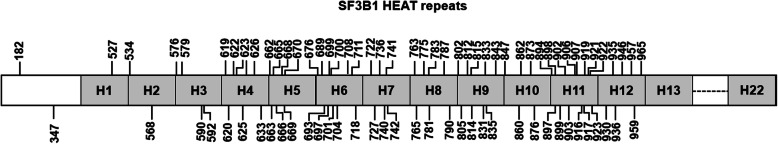
Distribution of mutations in *SF3B1*. More than 80 mutated codons have been found in the HEAT domain of the *SF3B1* gene, especially H4-H12

SF3B1 (splicing factor 3b subunit 1) is the largest subunit of the SF3B complex and functions by serving as a core component of the U2 snRNP, which is critical for branch site recognition and for the early stages of spliceosome assembly [49]. Structurally, the N-terminal hydrophilic region of SF3B1 has multiple U2AF2 binding motifs [50, 51], which may facilitate localization of the U2 snRNP to the vicinity of the branch site. Two-thirds of the C-terminus consists of 22 nonidentical HEAT (Huntingtin, elongation factor 3, subunit An of protein phosphatase 2A, and phosphatidylinositol 3-kinase (PI3K) target of rapamycin 1) repeats, which form rod-like helical structures, providing major scaffolding for the U2 snRNP to support interactions with other SF3B subunits, including p14 [52]. The HEAT-repeat superhelix of SF3B1 defines a composite RNA-binding platform for BS recognition. In human diseases, almost all mutations in *SF3B1* are located in the HEAT domain, particularly from H4-H12, as shown in Fig. 2. Among the mutated codons, 700 account for more than 50% of the variants observed, and additional codons (666, 662, 622, and 625) have been found to be hotspots for mutation [41, 53–55]*.*

### Mutant SF3B1 expression in cancer

Mutations in *SF3B1* have been implicated as common drivers of hematologic malignancies. Somatic *SF3B1* mutations are found in approximately 30% of patients with MDS and as many as 80% of patients with the MDS subtype characterized by ring sideroblasts (MDS-RARS) [41, 56, 57]*.* These mutations are also present in 20% of patients with myelodysplastic/myeloproliferative neoplasm (MDS/MPN) [41] and in 15% of patients with chronic myeloid leukemia (CLL) [58–60]*.* More recently, *SF3B1* mutations have been identified at relatively high frequency in some solid tumors, such as various pigmented tumors, including uveal melanoma (UM) [61, 62], mucosal melanoma [63], leptomeningeal melanoma [64] and blue nevus-like cutaneous melanoma [65], and neuroblastomas that arise following chromothripsis [66], estrogen receptor-positive breast cancers (BC) [67], pancreatic ductal adenocarcinoma [68], prostate cancer [69], prolactinomas [70], acute myeloid leukemia [71, 72], and many others [73–75].

### Prognostic value of SF3B1 mutation in cancer

The prognostic value of the *SF3B1* mutation in MDS remains controversial. Most studies have claimed that patients carrying an *SF3B1* mutation have a significantly better overall survival and a lower likelihood of their disease transforming into acute leukemia compared with patients without *SF3B1* mutations [11, 41, 76, 77]. In contrast, some studies found no significant effect of mutation on clinical outcomes [78, 79]. Regardless, *SF3B1* appears to be the only gene for which somatic mutations are associated with a good prognosis in MDS [41, 80]. As with MDS, *SF3B1* mutations were found to confer a favorable prognosis in uveal melanoma (UM), with a younger age of onset and concurrent disomy 3 [81, 82]. In addition, patients with *SF3B1*-mutated UM had better survival (at 5 years) than did *SF3B1* wild-type patients. Nonetheless, evidence also shows that the survival differences between patients with *SF3B1*-mutant tumors and *SF3B1* wild-type tumors are not significant over time, as indicated by follow-up data (at 10 years). Moreover, *SF3B1*-mutant UM is reported to cause late metastasis (median 8.2 years after initial diagnosis), suggesting that patients with *SF3B1* mutations are also at risk for metastasis, particularly late-onset metastasis [83]. It was inferred that the positive prognostic value of *SF3B1* mutation may be partly or completely lost after the acquisition of other gene mutations associated with disease progression [41]*.* In contrast to those in MDS or UM, *SF3B1* mutations that are cancer-related occur more commonly in advanced disease and tend to be associated with poor prognosis in other malignancies, including CLL. Thus, the prognostic relevance of *SF3B1* mutations in disease may be dependent on cellular contexts.

## Functional consequences of *SF3B1* mutation in cancer

### Multiple roles in tumor pathogenesis

To date, it remains unclear what functional role *SF3B1* mutations play in carcinogenesis, and it has not been well established whether deregulated SF3B1 activity is required for the maintenance of cancer [84]. To address these questions, the role of *SF3B1* mutations in malignant hematopoiesis has been investigated in vitro and in vivo. Regarding MDS, an *SF3B1*^*K700E*^ conditional knock-in mouse has been generated [85], and heterozygous expression of *SF3B1*^*K700E*^ caused progressive macrocytic anemia [85]. Moreover, *SF3B1*^K700E^ expression was associated with aberrant 3′ splice-site selection as well as increased nonsense-mediated decay [85]. In another study, conditional *SF3B1*^*flox-K700E/+*^ mice were generated by targeted modification of the *SF3B1* locus in JM8 mouse embryonic stem cells (ESCs). *SF3B1*^*K700E*/+^ mice develop progressive normocytic anemia without ring sideroblasts [86]. In addition to erythropoiesis, *SF3B1*^*K700E*/+^ mice had reduced numbers of hematopoietic stem cells (HSCs) and exhibited a myeloid cell bias [86]. Furthermore, the self-renewal potential of *SF3B1*^*K700E*/+^ HSCs was determined by their repopulating ability in competitive transplantation assays into either young or old recipient mice. The results revealed a fitness disadvantage of mutant over wild-type HSCs [86], which contrasts with observations that mutant *SF3B1* drives clonal hematopoiesis and may even be the sole identifiable driver mutation in human MDS [87, 88]. In addition, simultaneous expression of *SF3B1* and *SRSF2* mutations in mice resulted in increased apoptosis and reduced quiescence of hematopoietic stem progenitor cells (HSPCs). Moreover, combined expression of *SF3B1* and *SRSF2* mutations impaired expression of regulators of HSPC survival and increased sensitivity to inflammatory stimulation [89]. In human *SF3B1*-mutated CLL cases, ATM kinase function remained intact, and γH2AX formation, a marker for DNA damage, was found to be increased at baseline and upon irradiation, demonstrating that mutations in *SF3B1* are associated with increased DNA damage and/or an aberrant response to DNA damage [90]. In many cancer cells, *SF3B*1 mutation was found to dysregulate multiple cellular functions, including heme biosynthesis, immune infiltration, DNA damage response, R-loop formation, telomere maintenance, and Notch signaling [74, 91–93], as well as many cellular pathways, such as the mitochondrial, Notch and NF-κB pathways [91]. These results suggest that *SF3B1* mutations play multiple roles in the pathogenesis of tumors.

### Aberrant splicing events

**Fig. 3:**
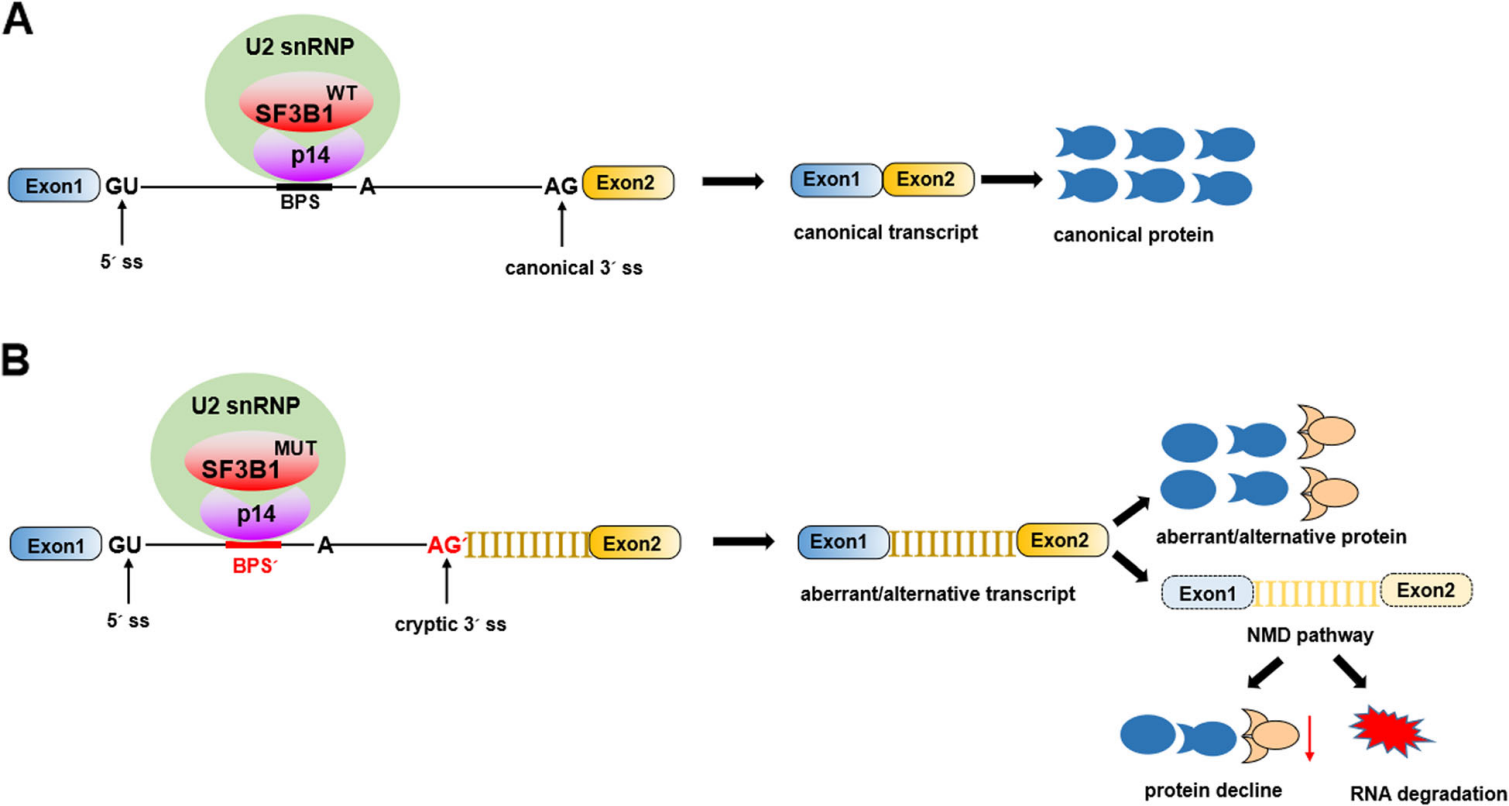
A model for aberrant 3′ ss selection induced by SF3B1 mutations. **a**: Schematic representation of SF3B1WT-mediated splicing, generating canonical transcript and producing canonical protein. **b**: Schematic representation of SF3B1MUT-mediated splicing, leading to the binding of U2 snRNP and alternative branchpoint sequence (BPSʹ) or usage of a cryptic 3′ splice and resulting in aberrant/alternative transcript with aberrant/alternative protein as well as increased nonsense-mediated decay (MMD)

**Fig. 4:**
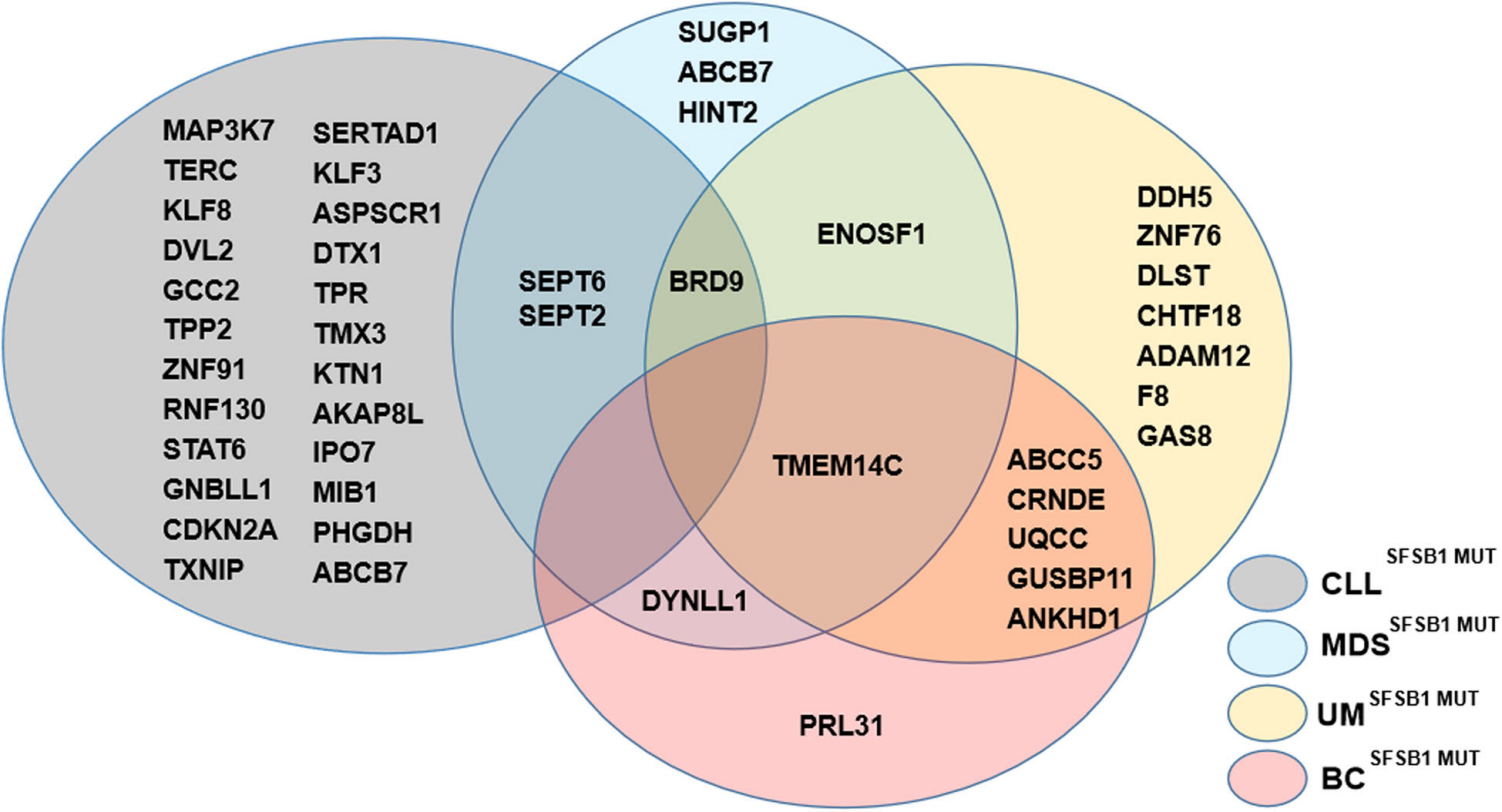
Venn diagrams showing *SF3B1* mutation-associated aberrant splicing genes identified by RNAseq and validated by qRT-PCR in CLL, MDS, UM, and BC. At least 46 genes are produced by aberrant splicing events in SF3B1^mut^ CLL, MDS, UM, and BC. Among them, *BRD9* is shared by CLL, MDS and UM and *TMEM14C* by MDS, UM and BC. *SEPT6* and *SEPT2* are shared by CLL and MDS, *ENOSF1* by MDS and UM, and *DYNLL1* by MDS and BC

In terms of SF3B1 as a core component of splicing machinery, it has been clearly proven that common and tumor-specific splicing aberrations are induced by *SF3B1* mutations, and aberrant 3′ ss selection has been established as the most frequent splicing defect [94], with increased alternative 3′ splice site usage [85] and usage of cryptic 3′ splice sites [86], as shown in Fig. 3. Strikingly, SF3B1 variants utilize a BPS that differs from that used by wild-type SF3B1 and requires the canonical 3′ ss to enable aberrant splicing during the second step [94]. *SF3B1* mutations result in neomorphic activity, causing hundreds of alterations both through aberrant splicing and dysregulated gene expression in common alternative splicing signatures in different types of cancers [95]. Furthermore, *SF3B1* mutations are linked to various RNA processing mechanisms, such as alternative terminal exons, alternative 3′ acceptor splice sites, alternative cassette exons, alternative first exon, alternative branch point usage, and intron retention [96, 97]. Approximately 50% of aberrantly spliced mRNAs are subjected to nonsense-mediated decay, resulting in downregulation of gene and protein expression [94, 98]. However, few of these genes have been functionally implicated in driving the diseases known to be associated with *SF3B1* mutations, including the genes shown in Fig. 4. Thus, the functional consequences and mechanisms of *SF3B1* mutations in cancers need to be further investigated.

#### Associations between aberrant splicing and clinical variables or patient survival

Fifteen aberrant splicing (*PARVG*, *RPRD1A*, *DOM3Z*, *CXXC1*, *AP1G2*, *SNRPN*, *TCEA2*, *NICN1*, *ABCC5*, *ERCC3, SNRPN*, *PPOX*, *GPR108*, *PSTPIP1*, *NICN1*) events have been correlated with clinical variables that showed a significant difference between *SF3B1*^*mut*^ and SF3B1^wt^ patients with MDS, including a lower percentage of bone marrow (BM) blasts and higher number of white blood cells, absolute neutrophil count (ANC), and platelet count (Plt) in the *SF3B1*^*mut*^ group [92]. Moreover, isoform expression of extracellular exosome/focal adhesion genes (*CRTC2*, *PPOX*, *AHSA2*, *DHP5*) produced by aberrant splicing events in *SF3B1*^*mut*^ patients has been identified as a significant survival predictor in MDS [92, 99]. The functions of eight genes (*BRD9*, *SUGP1*, *MAP3K*7, *TERC*, *KLF8*, *DVL2*, *SEPT2*, and *ABCB7*) with deregulated expression due to *SF3B1* mutations in tumors are discussed below.

#### BRD9

*Homo sapiens* bromodomain containing (BRD) 9 is a core component of the recently described mammalian BRG1-associated factor (BAF) chromatin remodeling complex that plays an important role in maintaining the transcriptional network of pluripotency [100]. Previous studies have reported that BRD9 is required for the survival of some cancer types, particularly cancers with mutations that affect polybromo-associated BAF and canonical BAF6 [101–103]. Recently, it was found that total levels of *BRD9* mRNA were reduced in patients with CLL, MDS and UM carrying *SF3B1* mutation. Further study found that mutant *SF3B1* suppressed levels of full-length BRD9 protein without generating a truncated BRD9 protein in UM (MEL270) or myeloid leukemia (K562) cells that express *SF3B1*^*K700E*^. Mutant *SF3B1* recognizes an aberrant, deep intronic branch point within *BRD9* and thereby induces the inclusion of a poison exon that is derived from an endogenous retroviral element, causing subsequent degradation of *BRD9* mRNA. Depletion of *BRD9* in turn causes loss of noncanonical BAF at CTCF-associated loci, resulting in alteration of BAF localization to chromatin. *BRD9* loss may also alter the expression of distinct genes involved in apoptosis and cell growth, adhesion and migration. In addition, it was also found that disruption of ncBAF-dependent regulation of HTRA1 (HtrA serine peptidase 1) contributes to the protumorigenic effects of *BRD9* loss. Correcting misspliced *BRD9* in *SF3B1*-mutant cells using antisense oligonucleotides or CRISPR-directed mutagenesis suppresses tumor growth [100]. These results implicate the disruption of noncanonical BAF in the diverse cancer types associated with *SF3B1* mutations and suggest a mechanism-based therapeutic approach for treating these malignancies [104].

#### SUGP1

SUGP1 (SURP And G-patch domain-containing protein, a member of the SURP family of splicing factors that likely interact with SF1 and RNA helicases) is also greatly reduced in samples from MDS patient harboring *SF3B1* mutation [53, 105–107]. Furthermore, loss or weakening of the interaction of SUGP1 with SF3B1 in the spliceosome was found to be the sole cause of defects in BP recognition, which results in the use of cryptic 3′ ss typically located 10–30 nt upstream of canonical 3′ ss [53]. That is, under normal conditions, the WT SF3B1 spliceosome uses a canonical BP and 30’ss for splicing; when the interaction between SF3B1 and SUGP1 is disrupted by *SF3B1* mutations, the mutant SF3B1 spliceosome uses an upstream BP and a cryptic 30′ ss for splicing. Furthermore, SUGP1 can associate with the mutant spliceosome and partially rescue splicing defects [53]. These data suggest that loss of *SUGP1* is a common defect in spliceosomes with cancer-associated *SF3B1* mutations and that the mutant spliceosome is “repairable” in principle via restoration of SUGP1 assembly [108].

#### MAP3K7

MAP3K7 (mitogen-activated protein kinase kinase kinase 7) encodes a kinase that mediates tumor necrosis factor α (TNFα), interleukin-1β (IL-1β), and Toll-like receptor signaling through the NF-κB, JNK, and MAPK pathways. Loss of *MAP3K7* results in the attenuation or promotion of inflammation, depending on the cellular context [109–111]. Reduced *MAP3K7* mRNA and protein are both found in isogenic cell lines and primary myeloid and lymphoid cells from MDS and CLL patients carrying *SF3B1* mutations [89]. In contrast, hyperactive NF-κB signaling with increasing p-p65 levels is detected in *SF3B1*^*K700E*^ human myeloid and lymphoid leukemia cells stimulated with LPS. Re-expression of *MAP3K7* in *SF3B1*^*K700E*^ cells leads to a significant decrease in p-p65 in both the resting state and following LPS exposure. Additionally, restoration of *MAP3K7* expression in *SF3B1*^*K700E*^ cells results in partial rescue of cell clonogenicity [89]. These data suggest that *SF3B1* mutations affect the splicing of *MAP3K7*, which, at least in part, the results in hyperactivation of NF-κB signaling, which is associated with MDS pathogenesis.

#### TERC

TERC (telomerase RNA component), encoding an essential RNA component of telomeres, plays a role in cell proliferative potential, telomerase activity and telomere length, and TERC mutations have been found in autosomal dominant dyskeratosis congenita (DC), aplastic anemia, MDS, and cervical cancer [112–115]. TERC is a noteworthy target with significantly increased expression in *SF3B1-*mutated CLL samples, as based on amplification using total RNA [91]. Upregulation of *TERC* and *TERT* (telomerase reverse transcriptase) was confirmed in Nalm6 isogenic cell lines with K700E and H622Q mutations. Not surprisingly, higher telomerase activity was detected in Nalm-6 *SF3B1*^*K700E*^ cells than in wild-type cell lines. These data suggest that mutant *SF3B1* may affect telomerase activity through dysregulation of *TERC* and *TERT* expression [91].

#### KLF8

KLF8 (Kruppel-like transcription factor 8) has been found to be involved in tumor cell proliferation, transformation, and progression and in DNA damage repair in several different tumors, including renal cell carcinoma, hepatocellular carcinoma and breast cancer [116]. KLF8 is consistently upregulated in *SF3B1-*mutated CLL samples at both single-cell and bulk RNA levels. Overexpression of *SF3B1* in cells induces phosphorylation of H2AX (γH2AX) [90, 91] and CHK2 (checkpoint kinase 2) [91], two markers of DNA damage, following exposure to gamma irradiation. These data demonstrate that single mutations in *SF3B1* are associated with increased DNA damage and/or an aberrant response to DNA damage and that *SF3B1* mutation-associated gene dysregulation is a contributor to altered DNA responses [90, 91]*.*

#### DVL2

Dishevelled 2 (DVL2) has been previously reported to play a role in both canonical and noncanonical Wnt signaling by binding to the cytoplasmic C-terminus of frizzled family members and inducing transduction of the Wnt signal to downstream effectors [117, 118]*.* Relatively high expression of the *DVL2* splice variant and the protein product of altered *DVL2* have been confirmed in individual primary CLL cells with *SF3B1* mutation [91]. Interestingly, altering *DVL2* does not influence Wnt signaling, but it does affect the Notch pathway: expression of altered *DVL2* markedly abrogates the activation of Notch1 and expression of the Notch pathway target gene *HES1* (hairy and enhancer of split-1). Moreover, combined expression of WT and altered *DVL2* reverses these suppressive effects, suggesting a dominant impact of altered DVL2 on the WT isoform [91]. These data implicate DVL2 as a target of mutated *SF3B1* through which alternative splicing modulates Notch signaling activity*.*

#### SEPT2

SEPT2 (septin 2), a member of the septin family of guanosine triphosphatases, plays a role in the biogenesis of polarized columnar-shaped epithelium by maintaining polyglutamylated microtubules and an important role in the regulation of mitosis and cell growth [119, 120]. SEPT2 is significantly downregulated in CD34^+^ cells with *SF3B1* mutations in patients with MDS [92]. In addition, the effect of *SEPT2* silencing on erythroid cell growth and differentiation has been studied in human BM CD34^+^ cells. It was found that *SEPT2* silencing leads to significantly impaired growth, G1/S transition arrest and a significant decrease in intermediate and late erythroid cell populations [92]. These results suggest that aberrant splicing of *SEPT2* may lead to impaired erythropoiesis in association with *SF3B1* mutations in patients with MDS.

#### ABCB7

ABCB7 (ATP binding cassette subfamily B member 7) encodes a half-transporter involved in the transport of heme from the mitochondria to the cytosol and plays a role in mitochondrial iron accumulation, isodicentric chromosome formation (X)(q13) and sideroblastic anemia, which is involved in many hematologic malignancies [121–123]. Aberrant splicing of ABCB7 occurs in MDS RARS and *SF3B1*^*mut*^ MDS patients and significant ABCB7 downregulation in *SF3B1*^*mut*^ cases [92, 96]. In normal bone marrow, *ABCB7* downregulation reduces erythroid differentiation, growth and colony formation and results in a gene expression pattern similar to that observed in intermediate MDS-RARS erythroblasts and in the accumulation of FTMT. Moreover, silencing *SF3B1* results in downregulation of *ABCB7* in K562 cells undergoing erythroid differentiation, implicating *ABCB7* in the acquisition of the RARS phenotype [124]. Furthermore, an *ABCB7* cryptic 3′ splice site event was detected in *SF3B1*-mutant HSCs carrying the *SF3B1*^*K700E*^ mutation; nonsense-mediated RNA decay (NMD) can target the aberrantly spliced *ABCB7* transcript and underlies the downregulation of *ABCB7* observed in MDS patients with *SF3B1* mutation. Moreover, treatment of *SF3B1*^*K700E*^*-*mutant cells with cycloheximide resulted in an increase in the aberrantly spliced form of the *ABCB7* transcript [96]. Interestingly, the sequence of the ABCB7 cryptic ss is not conserved in mice, and there is no aberrant splicing of ABCB7 in *SF3B1*-mutant murine cells [85, 86], which indicates significant differences in the transcripts affected because of *SF3B1*^*K700E*^ between humans and mice. These data provide strong evidence that *SF3B1* mutation leads to aberrant *ABCB7* splicing and downregulation via NMD in human cancer cells and suggest an ABCB7-based therapeutic approach for treating these malignancies.

In addition, the roles and mechanisms of many other key cancer-related genes that have been detected by RNA sequencing and validated by qRT-PCR in distinct types of cancer need to be further investigated, such as *TMEM14C* (transmembrane protein 14C), *SEPT6* (septin 6), and *ENOSF1* (enolase superfamily member 1), as shown in Fig. 4. More genes that can be categorized into one of two large groups, a cell-autonomous gene set and a set of genes in immune cells with signatures related to immune cell infiltration, need to be confirmed in vitro and in vivo [74].

### Changes in sensitivity to SF3B small-molecule inhibitors

Because the spliceosome SF3B complex has emerged as a potential therapeutic target, SF3B small-molecule inhibitors are currently under development and have entered clinical trials [125–127]. These inhibitors specifically target the SF3B protein complex, leading to the loss of spliceosome function with regard to 3ʹ splice site recognition and resulting in aberrant alternative splicing/mRNA transport. Among them, three bacterial fermentation products (FR901464, herboxidiene, and pladienolide) were identified as natural compounds with antitumorigenic properties [126]. In addition to natural drugs, meayamycin, E7107, and spliceostatin A (SSA) have been developed as synthetic analogs with improved stability and solubility [58]. Among them, pladienolide (Pla) stalls SF3B in an open conformation by acting like a wedge within a hinge, modulating the transition of SF3B to the closed conformation needed to form the BS adenosine-binding pocket and stably accommodate the BS/U2 duplex [128, 129]. Although Pla-B might be located in the vicinity of both SF3B1 and SF3B3, it only binds to SF3B3 [130]. Additionally, E7107, a synthetic derivative of pladienolide D, and SSA, a methylated derivative antitumor natural product FR901464, mainly destabilize U2 snRNP assembly at 3′ splice sites by blocking SF3B from binding to RNA [126]. E7107 targets SF3B1 to block ATP-dependent A complex formation as well as a conformational change in U2 that exposes the snRNA region responsible for base pairing to the branchpoint sequence [131], whereas SSA induces a conformational shift in the U2 snRNP to bind to “decoy” sequences that can occur upstream of the branchpoint sequence [58].

**Fig. 5:**
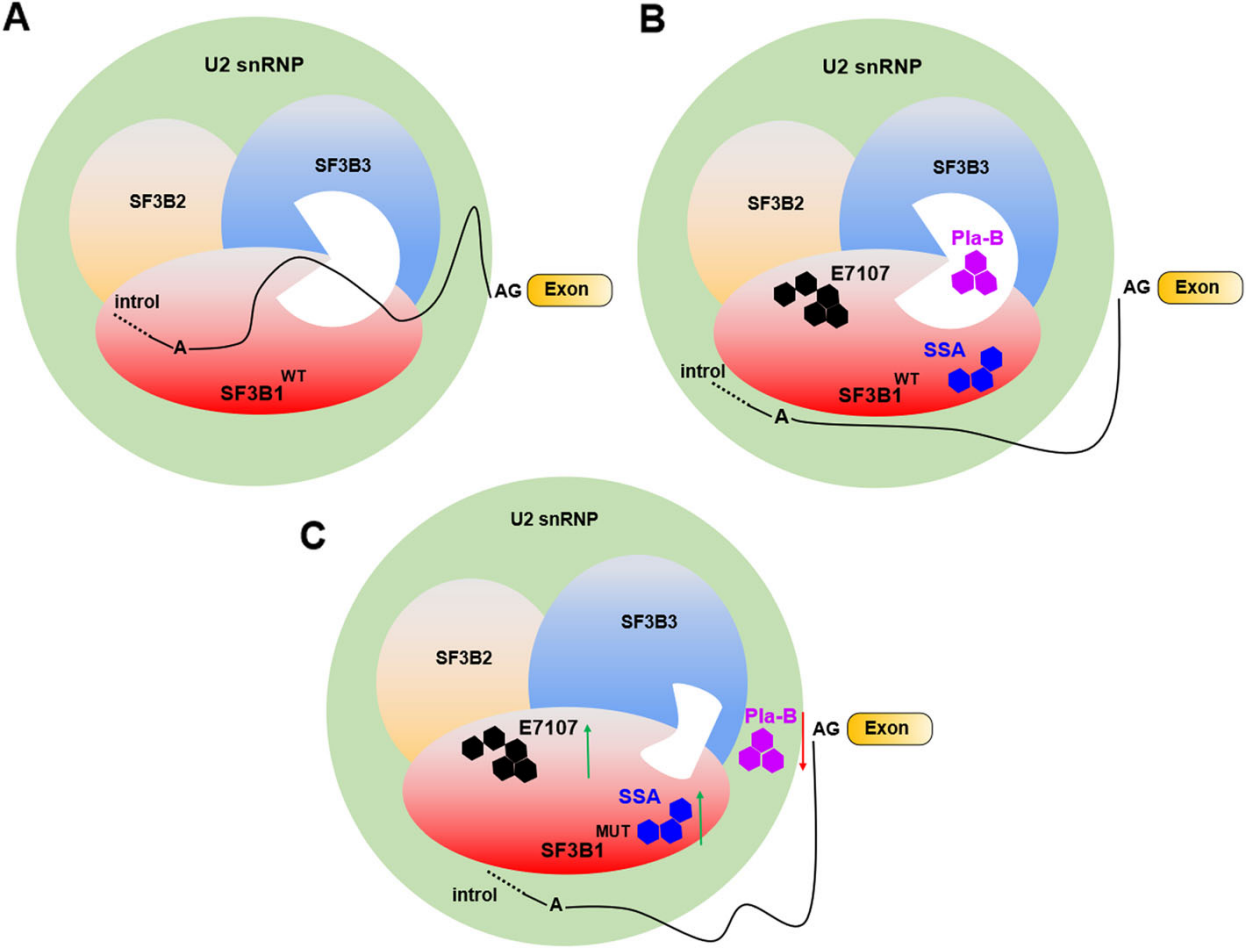
Molecular effects of SF3B small-molecule inhibitors on 3ʹ splice site recognition mediated by SF3B1 mutation. **a**: Normal condition of 3ʹ splice site recognition induced by SF3B1WT. **b**: Effects of inhibitors on 3ʹ splice site recognition induced by SF3B1WT. PlaB inhibits mRNA splicing by fitting into a space between SF3B1 and SF3B3; E7101 and SSA inhibit splicing by fitting into SF3B1. **c**: Effects of inhibitors on 3ʹ splice site recognition induced by SF3B1MUT. Mutations impair the binding of PlaB, E7101 or SSA to SF3B1

Interestingly, one disease-associated *SF3B1* mutation has the opposite effects on sensitivity to SF3B small-molecule inhibitors, as shown in Fig. 5. An R1074H mutation in *SF3B1* confers resistance to pladienolide activity by impairing the ability of pladienolide B to bind to SF3B due to the physical interference caused by *SF3B1* mutation [132], and it was speculated that *SF3B1* mutations in other HEAT repeats might have the same effect [58]. These data indicate that the mutant SF3B1 protein has an impaired response to pladienolide B. In contrast to pladienolide B, it was reported that the *SF3B1* mutant cell lines Panc 05.04 (pancreatic, K700E) and ESS-1 (endometrial, K666N) were more sensitive to SAA, leading to inhibition of cell growth [133]. In contrast to *SF3B1*^*+/+*^ murine HSPCs, *SF3B1*^*+/K700E*^ HSPCs were more sensitive to the spliceosome modulator E7107E [85]. Furthermore, treatment of *SF3B1*^*+/K700E*^ recipients with E7107 in vivo caused a significant decrease in CD45.2 chimerism in the peripheral blood, bone marrow, and spleen [85]. These data demonstrate that *SF3B1* mutation enhances the sensitivity of tumor cells to the spliceosome modulator E7107 and SAA but not pladienolide B. *SF3B1* mutation therefore sensitizes cells to pharmacologic targeting of wild-type SF3B1, consistent with the observation that the growth of *SF3B1*-mutant endometrial cancer and uveal melanoma cell lines was impaired by deletion of wild-type, but not mutant, *SF3B1* [84]. Although the mechanism of the enhancement sensitivity to splicing inhibitors is unknown, probably because a mutated *SF3B1* gene may be unable to tolerate further perturbations in splicing and therefore be preferentially sensitive to pharmacological splicing inhibition [134], these findings suggest that there may be a therapeutic window for the use of spliceosome modulators in the treatment of hematologic malignancies with *SF3B1* mutation [85].

Overall, mutated *SF3B1* has been detected in hematological malignancies and solid tumors and has been proven to be related to patient prognosis, abnormal transcription, alternative splicing, and sensitivity to SF3B small-molecule inhibitors. However, only a few genes affected by *SF3B1* mutation have been extensively studied to date, and the roles and mechanisms of these genes still need to be confirmed.

## SF3B1 or its mutation as a novel therapeutic target in cancer

Primary tumors with *SF3B1* mutations display alternative splicing in select key genes in cancer, including CLL, MDS, and uveal melanoma; this signature is conserved between cancer sites and is independent of the mutant amino acid [95], which implies that *SF3B1* mutations may represent an important clinical significance in tumors. Indeed, *SF3B1* is the most commonly mutated spliceosomal component gene in breast cancer, and mutations affecting this gene are significantly associated with ER-positive disease [67]. Moreover, *SF3B1* mutant cell lines were found to be sensitive to the SF3B complex inhibitor spliceostatin A, and treatment resulted in perturbation of the splicing signature [67]. Thus, given the multiple roles in the pathogenesis of tumors and splicing events induced by *SF3B1* mutations in cancer, as well as the potentially increased sensitivity of cancers to some SF3B small-molecule inhibitors, *SF3B1* or its mutation may represent a prognostic biomarker and therapeutic target for cancer, and pharmacological modulation of splicing may represent an important therapeutic strategy [134]. For example, although *SF3B1* mutations occur at a low frequency (1.1%) in prostate cancer (PCa) [69], *SF3B1* mRNA and protein levels are higher in tumor glands than in nontumor adjacent regions [135]. Notably, *SF3B1* expression correlated positively with clinical and molecular features, including Gleason score and vascular and perineural invasion. Pharmacological blockade of SF3B1 with pladienolide-B reduced malignant features of PCa cells and modulated key signaling pathways, malignancy markers, and expression of oncogenic splicing variants AR-v7 and In1-ghrelin, spliceosome components and splicing factors as well as expression of EJC and SURF components and NMD factors [135]. That is, dysregulation of SF3B1 expression may be involved in the development, progression, and aggressiveness of PCa, and SF3B1 might represent a new prognostic biomarker and therapeutic target in this devastating pathology. These results also indicate that SF3B1 inhibition leads to a decrease in the aggressiveness features of PCa cells through both direct and indirect mechanisms, possibly involving the modulation of different types of cellular stress processes. As SF3B1 variants exhibit an impaired response to pladienolide B [132], other inhibitors, such as E7107E and SSA, may have better antitumor activity for PCa. The prognostic and therapeutic potential of *SF3B1* in other types of cancer needs to be further studied.

## Conclusions and future perspectives

In eukaryotes, the RNA splicing system, as essential cellular machinery, is critical for successful transcription through constitutive splicing and guarantees the functional diversity of protein products through alternative splicing. Thus, deregulation of this machinery causes severe developmental abnormalities [42, 92, 136]. Recent studies have shown that mutations in the RNA splicing machinery significantly affect the RNA splicing system by altering many splicing patterns associated with 3′ splice sites, suggesting that aberrant splicing patterns induced by spliceosome mutations are directly linked to disease phenotypes [13]. SF3B1 is a shared core component of snRNPs both in major and minor spliceosomes and plays a critical role in the early and later stages of spliceosome assembly. *SF3B1* mutations occur in many types of tumor and play an important role in the development and progression of cancer. However, the functional impact and mechanisms of the *SF3B1* mutation-deregulated splicing pattern on oncogenesis need to be better understood. In addition, although it has become clear that aberrant premRNA alternative splicing is a major contributor to cancer phenotypes, studies on the misregulation of alternative splicing induced by *SF3B1* mutation in cancer have not kept pace with the latest data. Moreover, mutant *SF3B1* may have a distinct function not only in the direct regulation of RNA splicing but also in the elongation and stability of DNA, which may be important for the acquisition of specific disease phenotypes [36]. Further work is also required to evaluate the molecular mechanism by which mutations in *SF3B1* HEAT domains may influence the base-pairing potential of U2 snRNA [14]. Future clinical work is also needed to explore the relationship between *SF3B1* mutations and mutations in other cancer-related genes, including those of other spliceosome-associated proteins, splicing regulatory factors, and transcriptional factors. Moreover, a valuable tool for dissecting the effects of *SF3B1* mutations on the transformation, splicing, and functions of SF3B1 was established in a mouse model [87, 88], and investigation of the role of the yeast ortholog Hsh155 supports a novel mechanism in which SF3B1 helps to define the BS during premRNA splicing [137]. These findings are part of wide ongoing effort to generate genetically engineered model systems to study the biological and biochemical consequences of spliceosomal mutations in model systems as diverse as yeast, zebrafish, mouse, and human cells [138]. Finally, as the effects of splicing-modulation strategies targeting *SF3B1* mutations are currently unpredictable, laboratory, preclinical and clinical studies are required to understand the biological function and clinical significance of *SF3B1* mutations in cancer.

## Data Availability

Not applicable, all information in this review can be found in the reference list.

## Abbreviations

ABCB7: ATP-binding cassette subfamily B member 7
adRP: Autosomal dominant retinitis pigmentosa
ALS: Amyotrophic lateral sclerosis
BC: Breast cancer
BRD9: *Homo sapiens* bromodomain containing 9
BS: Branch site
CLL: Chronic myeloid leukemia
ENOSF1: Enolase superfamily member 1
ESE: Exonic splicing enhancer
ESS: Exonic splicing silencer
HSC: Hematopoietic stem cells
HSPC: Hematopoietic stem progenitor cell
hnRNP: Heterogeneous nuclear ribonucleoprotein
ISE: Intronic splicing enhancer
ISS: Intronic splicing silencer
KLF8: Kruppel-like transcription factor 8
MAP 3 K7: Mitogen-activated protein kinase kinase kinase 7
MDS: Myelodysplastic syndrome
snRNA: Small nuclear RNA
snRNP: Small nuclear ribonucleoprotein
SRSF2: Serine/arginine-rich splicing factor 2
SF3B1: Splicing factor 3b subunit 1
SURP1: SURP and G-Patch domain containing 1
TERC: Telomerase RNA component
TERT: Telomerase reverse transcriptase
TMEM14C: Transmembrane protein 14C
U2AF1: U2 small nuclear RNA auxiliary factor 1
UM: Uveal melanoma
ZRSR2: CCCH type zinc finger, RNA binding motif and serine/arginine rich protein 2
